# Programmable Bell state generation in an integrated thin film lithium niobate circuit

**DOI:** 10.1038/s41377-025-02150-z

**Published:** 2026-01-03

**Authors:** Andreas Maeder, Robert J. Chapman, Alessandra Sabatti, Giovanni Finco, Jost Kellner, Rachel Grange

**Affiliations:** ETH Zurich, Department of Physics, Institute for Quantum Electronics, Optical Nanomaterial Group, Zurich, Switzerland

**Keywords:** Quantum optics, Integrated optics

## Abstract

Entanglement is central to quantum technologies such as cryptography, sensing, and computing. Photon pairs generated via nonlinear optical processes are excellent for preparing entangled states due to their long coherence times and compatibility with fiber optic networks. Steady progress in nanofabrication has positioned lithium niobate-on-insulator (LNOI) as a leading platform for monolithic integration of photon pair sources into optical circuits, leveraging its strong second-order nonlinearity. Here, we present a reconfigurable photonic integrated circuit on LNOI, which combines two on-chip photon pair sources with programmable interferometers, enabling the generation of entangled states. The photon pair sources achieve a source brightness of 26 MHz nm^−1^mW^−1^ while maintaining a coincidence-to-accidental ratio above 100. We successfully interfere the two sources with 99.0 ± 0.7% visibility, demonstrating the indistinguishability required for producing entanglement on-chip. We show the preparation of any of the maximally entangled Bell states with fidelity above 90% verified by quantum state tomography. These results establish LNOI as a compelling, scalable platform to explore integrated quantum photonic technologies enabled by high-brightness sources of entangled quantum states.

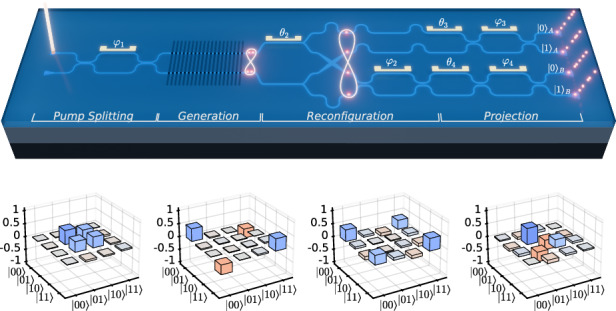

## Abstract (web summary)

We demonstrate a programmable thin-film lithium niobate photonic circuit capable of high-brightness generation and reconfigurable projection of path-encoded Bell states with high fidelity.

## Introduction

Photonic systems are at the forefront of research in quantum technologies for applications like quantum computing, quantum cryptography, or quantum sensing^1^. These applications benefit from the intrinsic advantages of photons, such as their low decoherence, ease of long-distance transmission through optical fiber, and weak coupling to the environment. Entanglement is the key requirement for quantum error correction, quantum secure communication, and quantum computational advantage for classically intractable tasks like boson sampling. Therefore, a scalable, efficient, stable, and compact source of entangled photons is one of the key requirements for advancing quantum information science.

Fundamental demonstrations of quantum optics and its applications in technology have been based on free-space or fiber-based experiments, which use spontaneous parametric downconversion (SPDC) as a source of entangled photons. This process relies on the second-order nonlinearity (*χ*^(2)^) of non-centrosymmetric crystals to generate photon pairs entangled in polarization, frequency, space, or time^2–5^. Among the most common *χ*^(2)^-crystals is lithium niobate (LN), which was used for experimental realizations of high fidelity entangled states^6^, generation of squeezed light^7^, quantum key distribution^8^, and quantum teleportation^9^. Although these LN pair sources offer the required high brightness, the experiments require phase stabilization and bulky optical elements for the transformation of the generated quantum state, which makes the approach inherently non-scalable.

Integrated photonics is a scalable solution for quantum photonic technologies, with demonstrations of unprecedented complexity in recent years^10,11^. Unlike free-space implementations, which commonly exploit entanglement in the polarization degree of freedom, integrated photonics predominantly relies on spatial encoding of photons. In this encoding, quantum operations can be realized using on-chip programmable interferometers^12^, as has been demonstrated, for example, in silicon, silicon nitride, or silica photonics^13–16^.

The development of lithium niobate-on-insulator (LNOI) wafers enables the combination of the strong *χ*^(2)^ nonlinearity of LN with low-loss optical waveguides that support tight bending radii and scalable fabrication processes^17^, making it an ideal platform for exploring integrated quantum photonics. Numerous static and dynamic building blocks for classical applications have been realized on this platform^18–21^. For quantum photonics, realizations of high brightness sources^22^, on-chip two-photon interference^23–25^, and compatibility with superconducting single photon detectors in cryogenic environments^26,27^ represent essential developments. Despite these achievements, most research on LNOI has remained limited to isolated devices or simple assemblies of a few elements, rather than fully integrated quantum circuits. Moreover, architectures that bring together high-brightness on-chip sources and reconfigurable circuits remain unexplored in LNOI.

This work combines high-performance building blocks developed on the LNOI platform into a single, programmable circuit that generates entangled Bell states on-chip. We monolithically integrate a pair of periodically poled LNOI waveguides with a reconfigurable interferometric circuit to realize path-encoded Bell states. The photon pair sources have an on-chip spectral brightness of 26 MHz mW^−1^ nm^−1^, and we observe 99.0 ± 0.7% two-photon interference visibility. We verify the presence of on-chip generated states by quantum state tomography, which allows us to reconstruct the full density matrix of the state. The state projections required for quantum state tomography are performed directly on-chip, making the approach inherently phase-stable. Through additional control of phases, we are able to reconfigure the circuit to generate different two-qubit states. We demonstrate the generation of computational basis states with fidelities above 95% and entangled Bell states with fidelities above 90%. These results mark an important step toward scalable quantum photonic systems leveraging *χ*^(2)^-sources. By combining many building blocks into a single, programmable device, this work showcases the potential of the LNOI platform for realizing advanced quantum information processing circuits.

## Results

### Device Principle

**Fig. 1:**
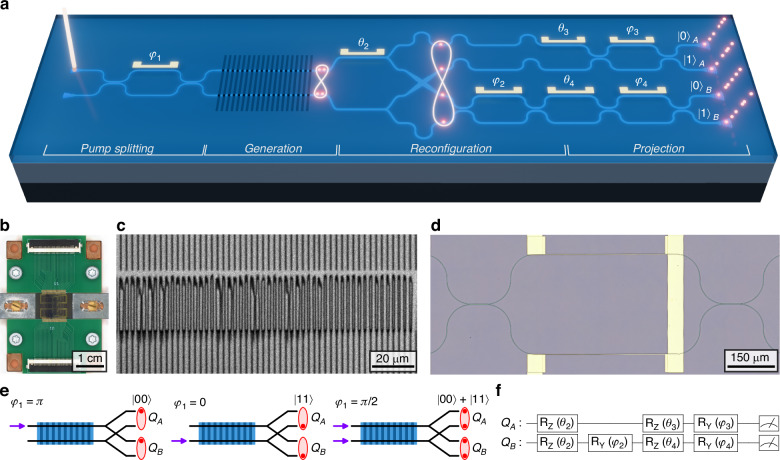
Working principle and fabrication result of LNOI circuit. **a** CW pump light (*λ*_*p*_ = 775 nm) is coupled to the chip via grating couplers and split using a Mach-Zehnder interferometer (MZI). This generates a superposition of photon pairs in two periodically poled waveguides, which is split into two dual-rail encoded qubits, creating an entangled Bell state. Integrated thermo-optic phase shifters and MZIs are used to transform and project the generated two-qubit state. **b** Image of the final photonic integrated circuit, including electrical packaging. **c** Two-photon microscopy image of periodically poled region prior to waveguide etching. **d** Microscope image of an integrated MZI with thermo-optic phase shifter. **e** Different pumping scheme generating different states dependent on the pump phase *φ*_1_. **f** Equivalent quantum circuit representation in the two-qubit picture

Figure 1a shows a schematic of the LNOI circuit, and an image of the fabricated and electrically packaged device is shown in Fig. 1b. It combines two 1.5 mm-long waveguide-integrated SPDC sources enabled by local periodic poling of the LN film. This periodic inversion of the *χ*^(2)^ coefficient (see Fig. 1c) implements quasi-phase matching, compensating the momentum mismatch between pump, signal, and idler waves, ensuring efficient photon pair generation^28^. By pumping both waveguides at *λ*_*p*_ = 775 nm, signal and idler photons centered around the degenerate wavelength *λ*_*s*_ = *λ*_*i*_ = 1550 nm are created. Importantly, because the sources are pumped with a continuous wave (CW) laser, at any given time, only a single photon pair is generated as a superposition of emission from the two sources. A Mach-Zehnder interferometer (MZI, see Fig. 1d) prior to the sources allows for controlling the relative pump power by adjusting its phase difference *φ*_1_. After the poled waveguides, the photon pairs are probabilistically split into two dual-rail encoded qubits *Q*_*A*_ and *Q*_*B*_ using two Y-splitters and a waveguide crossing. As shown in Fig. 1e, the pumping scheme can be adjusted to produce either pure computational basis states $| 00\rangle$ or $| 11\rangle$ (*φ*_1_ = 0 or *π*) or a maximally entangled Bell state $| {\Phi }^{+}\rangle \propto | 00\rangle +| 11\rangle$ (*φ*_1_ = *π*/2). Two additional phase shifters controlling *θ*_2_ and *φ*_2_ (see Fig. 1a) are used to modify the generated state further. If equal pumping with *φ*_1_ = *π*/2 and equal source efficiency *η*_*A*_ = *η*_*B*_ = 1 are assumed, the state can be rewritten in Bell basis as

1 $$\begin{array}{rcl}| \psi \rangle & \propto & i\sin ({\theta }_{2})\sin (\frac{{\varphi }_{2}}{2})| {\Phi }^{+}\rangle \\ & & +\cos ({\theta }_{2})\sin (\frac{{\varphi }_{2}}{2})| {\Phi }^{-}\rangle \\ & & +\cos ({\theta }_{2})\cos (\frac{{\varphi }_{2}}{2})| {\Psi }^{+}\rangle \\ & & +i\sin ({\theta }_{2})\cos (\frac{{\varphi }_{2}}{2})| {\Psi }^{-}\rangle \end{array}$$

where global phase factors and normalization constants have been omitted. This establishes that *φ*_2_ dictates whether the odd or even parity Bell states, $| {\Psi }^{\pm }\rangle$ or $| {\Phi }^{\pm }\rangle$, respectively, are being generated, while *θ*_2_ controls the relative symmetry of the resulting state. Given the control over the phases through thermo-optic (TO) phase shifters, the circuit can be reconfigured to generate any one of the four Bell states. Additionally, in the case where only one source is pumped, the phases can be programmed to prepare the other two computational basis states $| 01\rangle$ and $| 10\rangle$. A more extensive theoretical derivation of the state generation is given in the supplementary material.

The second half of the circuit shown in Fig. 1a facilitates quantum state tomography to reconstruct the full density matrix of the state generated on-chip. For this, an additional MZI (phases *φ*_3_, *φ*_4_) with an external phase shifter (phases *θ*_3_, *θ*_4_) is added to each qubit. This applies unitary transformations to the qubits, which are used to implement the required projections, as illustrated in Fig. 1f with the quantum circuit diagram realized by the LNOI circuit. As for all the previous phases, the tomography phases are physically controlled using TO phase shifters (see Fig. 1d). Coupling of the pump laser to the circuit, as well as the generated photons to a single photon detection system, is facilitated through grating couplers. More details on the device fabrication can be found in the Methods section and on the characterization of building blocks in the supplementary material.

### Photon pair sources

The high *χ*^(2)^ nonlinearity of LNOI enables efficient photon pair generation via SPDC. By etching waveguides in regions where the LN film has been periodically poled, high brightness integrated photon pair and heralded single photon sources can be realized^22^. A two-photon microscope image of the inverted domains, acquired prior to waveguide etching, is shown in Fig. 1c (see Methods for fabrication details).

**Fig. 2:**
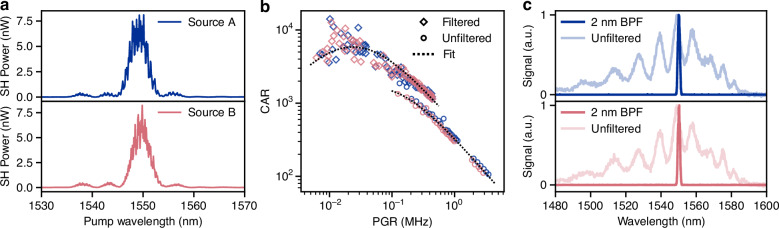
Characterization measurements of calibration photon pair sources. **a** Second harmonic (SH) spectra for two periodically poled waveguide, source A and B, respectively. **b** Coincidence-to-accidental ratio (CAR) as a function of pair generation rate (PGR) for both sources. Circle markers are measurements without spectral filtering, diamond markers are measurements including a 2 nm bandpass filter (BPF). **c** Time-of-flight spectroscopy measurements of top and bottom pair source with and without the BPF

For quantitative characterization of the SPDC sources, we fabricated a pair of 1.5 mm-long calibration waveguides in an identical periodically poled region separate from the full circuit. Figure 2a shows the measured second harmonic signal generated by calibration sources A and B. We observe very good spectral overlap between the two second harmonic intensities, and a phase-matching wavelength close to 1550 nm as targeted in the design. The normalized second harmonic conversion efficiencies of the two sources are 2150% W^−1^ cm^−2^ and 2708% W^−1^ cm^−2^, respectively, which are close to the theoretical limit of 3300% W^−1^ cm^−2^. The difference in efficiency between the two sources is believed to be due to the spectral response of the output grating (see supplementary material).

Having established near identical phase matching and second harmonic generation efficiency, we characterize the SPDC process by probabilistically splitting the photon pairs with an off-chip fiber-beamsplitter and performing coincidence measurements between the two outputs. We compare photon pairs that are not spectrally filtered beyond the response of the grating couplers to those passing through a 2 nm bandpass filter. Figure 2b presents the on-chip pair generation rate (PGR) and coincidence-to-accidental ratio (CAR) for both sources. The on-chip pump powers in these measurements vary between 0.1 and 10 *μ*W for both filtered and unfiltered cases. The CAR follows the expected PGR^−^^1^ dependence, with the expected decrease at low rates (dashed lines in Fig. 2b, see supplementary material for more details)^29^.

The on-chip source brightness of the unfiltered sources is estimated to be 1.5 GHz mW^−1^ and 1.7 GHz mW^−1^ for sources A and B, respectively. The bandpass filter reduces the source brightness to 51.6 and 51.3 MHz mW^−1^, respectively. This corresponds to a spectral brightness of approximately 26 MHz nm^−1^ mW^−1^ for both sources. These values compare well to existing literature on SPDC in periodically poled LNOI waveguides^22^, and outperform similar photon pair sources based on third-order nonlinearity in silicon waveguides^30,31^. A more detailed comparison is given in the supplementary material.

To confirm the spectral properties of the generated photons, we used a time-of-flight technique to measure the photon spectra directly (see Methods). The single photon spectra measured with and without a bandpass filter are shown in Fig. 2c. The unfiltered photons show a broad spectrum of around 100 nm bandwidth, which is expected for the type-0 SPDC process used here. It is limited by the spectral bandwidth of the grating couplers used to couple the photons to the fiber. The filtered photons inherit the spectrum from the bandpass filter as expected.

Overall, these results demonstrate the high efficiency of the on-chip SPDC photon pair sources. The excellent spectral overlap and matched performance of the two sources are essential for high-visibility quantum interference, forming the foundation for the generation of entangled states.

### Calibration and Tomography measurements

**Fig. 3:**
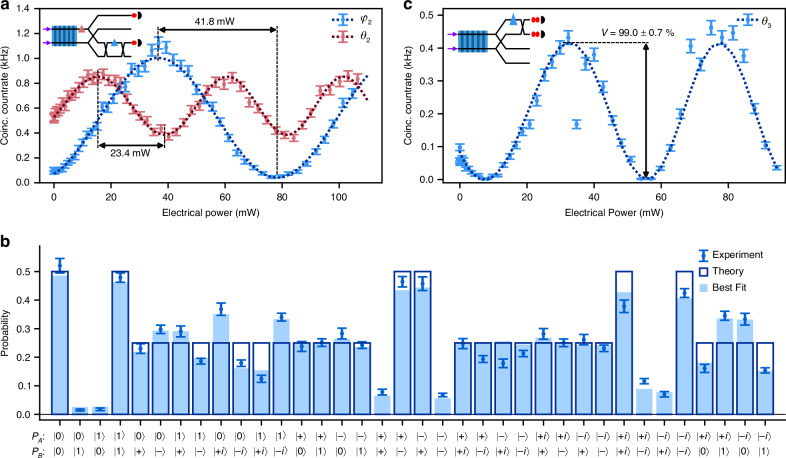
Interference characterization and projection measurements. **a** Calibration measurements for thermo-optic phase shifters of reconfiguration phases *φ*_2_ and *θ*_2_. Dotted lines are calibration fits. The inset indicates simplified circuit diagram. **b** Overcomplete set of projections used for quantum state tomography of the $| {\Phi }^{+}\rangle$ state. Experimentally measured probability is compared to the theoretically expected results and the obtained best fit using a global minimization algorithm. **c** Two-photon interference measurement using pairs from independent sources with 99.0 ± 0.7% visibility *V*. The inset represents a simplified schematic of the experiment

Accurate operation of the programmable circuit relies on calibration of the TO phase shifters. To achieve this, we determine the phase-voltage relationship of each phase shifter individually. Calibration curves for the state reconfiguration phases *φ*_2_ and *θ*_2_ are shown in Fig. 3a as a function of the dissipated electrical power. A sinusoidal fit to the data provides a model of the voltage-phase relationship, which after inversion enables us to determine the required voltage for a target phase. Note that countrate for the *θ*_2_ calibration oscillates at roughly twice the frequency, which is consistent with the theoretical prediction in eq. (1). Each calibration is performed with other phase shifters turned off as much as possible to minimize cross-talk effects. Therefore, the single photon visibilities in Fig. 3a are not maximized. Moreover, for most of the calibration procedure, the pump phase is set to *φ*_1_ = *π*/2, which assumes identical source efficiencies. However, two-photon interference measurements discussed later (see Fig. 3c) showed that balanced pumping of the sources required *φ*_1_ ~ *π*/3 to compensate for a mismatch in SPDC generation probability between the two sources.

As mentioned in the working principle, the circuit allows for performing quantum state tomography to measure the full density matrix of generated quantum states. The tomographic reconstruction is performed using a complete set of measurements with projections onto the single-qubit Pauli eigenstates $\{| 0\rangle ,| 1\rangle ,| +\rangle ,| -\rangle ,| i\rangle ,| -i\rangle \}$ for each qubit, yielding 6^2^ = 36 measurements. The measurements are performed by setting the projection phases to implement the qubit rotations and collecting coincidence counts over 2 s. This short integration time is enabled by the high brightness sources leading to an off-chip PGR of around 1 kHz during the tomography experiments. In addition, parallel detection of all four qubit rails reduces the acquisition time to 30 s per state tomography. We perform maximum likelihood estimation using the 36 coincidence counts to reconstruct the full quantum state^32^. More details on the state reconstruction can be found in the supplementary material.

To illustrate the results of the projection measurements, Fig. 3b shows the measured probabilities for each projection of a $| {\Phi }^{+}\rangle$ state. Already from the Pauli-*Z* basis projections, one can identify the characteristic superposition of $| 00\rangle$ and $| 11\rangle$. All of the experimental results show good agreement with theoretically expected probabilities. The density matrix reconstructed via maximum likelihood estimation shows good agreement to the experimental and theoretical probabilities. These results validate the functionality of the reconfigurable on-chip projections based on TO phase shifters and support their use in more complex quantum circuit on the LNOI platform.

### Entangled state generation

Stable and scalable generation of entangled photon pairs is a key requirement for photonic quantum technologies. In our integrated LNOI circuit, we use the probabilistic nature of SPDC to create path entangled states. The programmability enables the preparation of different entangled states, making it a versatile source for various quantum protocols.

Given that the photon splitting by the Y-splitters is entirely probabilistic, we can first investigate an entangled two-mode state. It is generated when both sources are pumped, but the photon pair is not split (see inset of Fig. 3c). The state generated in that case, a *N*00*N* state with *N* = 2, can be investigated when configuring the subsequent MZI as a 50:50 beam splitter (*φ*_3_ = *π*/2). In this configuration, we can observe a two-photon interference effect referred to as the time-reversed Hong-Ou-Mandel effect^24,33^. With the phase *θ*_3_ we can control whether the photon pairs interfere constructively or destructively, which we report in Fig. 3c. The observed visibility of 99.0 ± 0.7%, obtained from the maximum of the sinusoidal fit and the minimum measured countrate, indicates that photon pairs from separate sources are in fact indistinguishable to a very high degree.

Having shown the capability of generating entangled two-photons states with only part of the circuit, we next demonstrate on-chip generation of eight different two-qubit states. For this, we pump the circuit with 150 *μ*W CW laser light and measure coincidence counts between the four combinations of qubit rails. During this experiment, we observed an off-chip PGR of around 1 kHz and the CAR around 100. Due to insertion loss of additional on-chip components, and less than optimal fiber-to-chip coupling due to usage of fiber arrays instead of single fibers, these values are lower than the ones reported for the calibration source characterization in Fig. 2. However, they were sufficient to conduct the experiment without the need for excessive integration time.

**Fig. 4:**
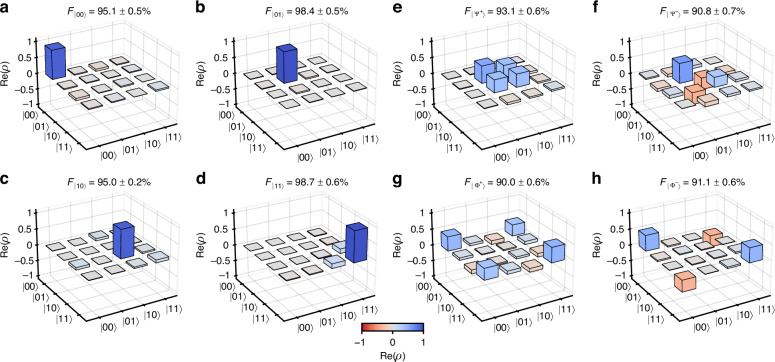
Reconstructed Density Matrices. Real part of density matrices obtained through maximum likelihood estimation for **a** $| 00\rangle$, **b** $| 01\rangle$, **c** $| 10\rangle$, **d** $| 11\rangle$, **e** $| {\Psi }^{+}\rangle$, **f** $| {\Psi }^{-}\rangle$, **g** $| {\Phi }^{+}\rangle$, and **h** $| {\Phi }^{-}\rangle$ state. ${F}_{| \psi \rangle }$ indicates the fidelity of the shown density matrix to the respective target state $| \psi \rangle$. The imaginary parts are given in the supplementary material

The programmability of the circuit is first demonstrated by preparing all four computational basis states, via pumping a single source only (see Fig. 1e) and using *φ*_2_ to flip the second qubit. The real parts of the reconstructed density matrices are shown in Fig. 4a–d. All of them show high fidelity above 95% to the respective target state. Next, both sources are pumped with *φ*_1_ = *π*/3, to achieve equal pair generation probability in both waveguides. Reconfiguration of *φ*_2_ and *θ*_2_ allows us to generate each of the four Bell states. Their reconstructed density matrices are shown in Fig. 4e–h and have fidelities of above 90%, with $| {\Psi }^{+}\rangle$ achieving the highest fidelity of 93.1 ± 0.6%. Besides fidelity, we also report the concurrence *C* of the states, which is computed from the density matrices. For the highest fidelity state $| {\Psi }^{+}\rangle$, we find *C* = 0.80 ± 0.03, which is above the limit $C=1/\sqrt{2}$ required to guarantee violation of the Clauser-Horne-Shimony-Holt (CHSH) inequality^34^. Notably, all four Bell states satisfy this criterion. Furthermore, we compute the von Neumann entropy *S*_*A*_ and *S*_*B*_ of each qubit by tracing out the other qubit. Those values vary between 0.64 and 0.69, which match well with the theoretical value $\ln (2)$ for a maximally entangled state in two dimensions. A full table of calculated state metrics is available in the supplementary material.

All of the findings show that the integrated circuit is capable of generating entangled two-photon states directly on-chip. Moreover, the inherent phase stability of integrated photonics is leveraged by including projections required for quantum state tomography on-chip as well. The ability to prepare a complete set of Bell states at high generation rates above 1 kHz highlights the potential of this LNOI circuit as a compact, programmable source of entangled states for quantum photonic applications.

## Discussion

In conclusion, we have demonstrated a programmable monolithic LNOI photonic integrated circuit capable of on-chip generation of entangled two-photon states. By combining two independent SPDC photon pair sources, each achieving an on-chip spectral brightness of 26 MHz nm^−1^ mW^−1^, with reconfigurable interferometric elements, we first prepared *N*00*N* states which show time-reversed Hong-Ou-Mandel interference with a visibility of 99.0 ± 0.7%. This confirms the high degree of indistinguishability of the independent SPDC sources. Using the full circuit, we realized two-qubit states and performed tomographic reconstruction of the density matrices using on-chip projections and only a few seconds of integration time per measurement. The achieved fidelities exceed 95% for computational basis states and 90% for each of the four Bell states. Analysis of concurrence and von Neumann entropy confirms that the Bell states violate the CHSH inequality and are maximally entangled with highly mixed subsystems.

Similar experiments have been realized on silicon photonics^35–37^, and rely on spontaneous four wave mixing (SFWM) photon pair sources (see supplementary material). These require the use of pulsed lasers and cm-long waveguide spirals to operate at reasonable PGR, and are fundamentally limited by two-photon absorption^38^. Other realizations of reconfigurable interferometric circuits rely on off-chip *χ*^(2)^ sources or cryogenically operated quantum dots for state generation, which leads to additional coupling loss, limiting the achievable state generation rates^39,40^. While recent work has demonstrated SPDC sources in silicon nitride using all-optical poling^41^, its source brightness remains considerably lower than achieved here, underscoring the benefit of the intrinsic *χ*^(2)^ nonlinearity of LN. Our experimentally achieved off-chip PGR demonstrates that integrated SPDC sources on the LNOI platform can outperform SFWM-based sources, while requiring only mm-long straight waveguides and CW pump lasers. Considering that our circuit currently suffers from photon loss at the fiber-to-chip interface, there is significant room for improvement of the PGR. This only marks a technical challenge, seeing that efficient fiber-to-chip couplers with around 1 dB loss per grating can be achieved on the LNOI platform^20,42^. Moreover, we envision to combine such a circuit with waveguide-integrated single-photon detectors in the future, which would significantly improve the detection rates. Furthermore, the measured two-photon interference visibility is equally high with reported SFWM-based chips^35,43^, which shows that high degrees of photon indistinguishability is achievable with periodically poled waveguides on the LNOI platform. Fabricating more waveguides in the same poled region offers a direct path to scaling to multi-qubit states. The close agreement of phase-matching wavelengths across multiple devices highlights that fabrication challenges can be effectively managed with precise calibration. We anticipate that the superior brightness will be especially advantageous, which will be critical when realizing higher photon number states like Greenberger-Horne-Zeilinger states^40,44^.

This work provides a first demonstration of combining high-performance linear building blocks developed on the LNOI platform with on-chip SPDC sources into a single, programmable quantum photonic circuit. While LNOI shares common challenges, such as thermal management, fiber-to-chip coupling and co-integration with pump lasers, with other integrated photonic platforms, continued efforts to scale LNOI fabrication are expected to further enhance the platform, supporting the development of more advanced and scalable quantum photonic circuits.

## Materials and Methods

### Device Fabrication

The integrated circuit was fabricated on a 300 nm x-cut LNOI chip with 4.7 *μ*m silicon dioxide bottom oxide layer. First, 1.5 mm long and 30 *μ*m wide regions of periodically poled LN film are created by applying high voltage pulses to comb-like electrodes. The poling period used is *Λ* = 2.87 *μ*m and was tailored to the local LN film thickness at the poling site, which was measured with optical reflectometry. This step was crucial to ensure consistent phase matching across the chip, as the phase matching wavelength is highly sensitive to film thickness variations. After poling, LN waveguides are patterned using electron-beam lithography and Argon ion milling in an inductively coupled plasma reactive ion etching tool. The etch depth of 200 nm is controlled using an end-pointing system. A cladding layer of 1 *μ*m silicon dioxide is deposited on the patterned chip, followed by fabrication of the gold TO electrodes using electron beam lithography and a standard double-layer liftoff process. The TO phase shifters have a low footprint of 1 *μ*m × 100 nm × 0.4 mm. For electrical connections to the phase shifters, a 300 nm thick set of gold routing electrodes is deposited. These electrodes are wirebonded to a printed circuit board mounted together with the photonic chip on a copper mount, which is held at 22 ^∘^C with a Peltier element during all the measurements.

### Twin Photon Pair Source Measurements

For measurements of the on-chip source properties, we use a pair of poled waveguides in a domain inversion region with the same parameters as the one in the reconfigurable circuit. This enables direct measurements of source properties without parasitic effects from the subsequent on-chip components. Each source includes an on-chip wavelength division multiplexer (WDM) to separate light around 775 (NIR) and 1550 nm (IR). We use cleaved single-mode fiber to couple to the sources with monolithically integrated grating couplers designed for the respective wavelength. For the second harmonic measurement in Fig. 2a we sweep a tunable CW laser around 1550 nm and synchronously measure at the NIR output port with a power meter. An additional off-chip WDM is used for filtering out any residual pump. For the SPDC measurements, we couple a 775 nm CW laser through the NIR port of the on-chip WDM and collect the photon pairs through the IR gratings. We use a fiber-based long-pass filter for pump rejection filtering, a 50:50 fiber beamsplitter for probabilistic splitting of the photon pairs, and two superconducting nanowire single photon detectors (SNSPDs) to measure the photon counts. A time tagging unit is used to measure correlations between the signal and idler ports. Additionally, we use manual polarization controllers to control the polarization of the input light as well as the output to optimize the SNSPD detection efficiency. As described in the main text, for some measurements, a fiber-based bandpass filter is added before splitting the photons.

The photon spectra in Fig. 2c are measured using time-of-flight single photon spectroscopy^45^. A dispersion compensation module with a time dispersion of 0.5 ns nm^−1^ is added in the idler path after the beamsplitter, which introduces a wavelength-dependent delay to the idler photon. This stretches the temporal correlation histogram. Using the known bandpass spectrum and dispersion characteristics of the module, the spectral properties of the idler photon is reconstructed.

### Bell state generator measurements

For measurements with the full device, we use a cleaved single-mode fiber to couple light at 775 nm through the input grating. The polarization is controlled using an off-chip polarization controller optimized for maximum signal. For collecting the signal, we use an 8-channel fiber array, allowing us to couple all four outputs simultaneously. They are subsequently connected to four independent SNSPDs, each with an additional polarization controller to maximize the detection efficiency. Two bandpass filters are used on the outputs of *Q*_*A*_, which effectively filter *Q*_*B*_ as well since we post-select on measurements with one photon per qubit. This filtering increases the photon coherence length and reduces the impact of small path length mismatches between the two qubit rails, which is present due to a design oversight. Additionally, this avoids any issues with the spectral response of on-chip components like directional couplers. A time tagging unit is used to measure the correlation histogram between all combinations of the output ports. The TO phase shifters are electrically controlled using a programmable multi-channel voltage source. The phase-voltage relationship is modeled as

$$\xi (V)={\xi }_{0}+\frac{\alpha {V}^{2}}{1+\beta {V}^{2}}$$

where the random phase offset *ξ*_0_ and the parameters *α* and *β* are obtained through fitting of calibration curves for each individual phase shifter. This relationship is inverted to get the voltage required to apply a target phase. During the tomography measurements, the phase shifters consume an average electrical power of 240 mW.

### Maximum likelihood estimation

To reconstruct the two-qubit quantum states from measured data, we employed maximum likelihood estimation of the density matrix *ρ*^32,35^. The likelihood function ${\mathcal{L}}(\rho )$ was defined based on the least squares difference between observed coincidence counts and the theoretical expectation value of the set of projection measurements. To enforce the physical constraints of *ρ*, we parametrized it as *ρ* = *T*^†^*T*/*Tr*(*T*^†^*T*), where *T* is a lower-triangular complex matrix^32^. In addition to the 16 free parameters defining *T*, we also use four normalization parameters capturing the efficiency difference between the SNSPDs used. $\log [{\mathcal{L}}(\rho )]$ was then minimized numerically over the 20 free parameters using a global optimizer. The density matrix ${\rho }_{\min }$, minimizing the likelihood function, was used to compute relevant figures of merit such as fidelity and concurrence. Statistical uncertainties are estimated through Monte Carlo resampling of the experimentally obtained counts, assuming Poissonian distribution. The entire procedure is described in more detail in the supplementary material.

## Supplementary information

Supplementary Material

## Data Availability

Raw data and evaluation code are available from the authors upon reasonable request.
