# Supplementary material for: Programmable Bell state generation in an integrated thin film lithium niobate circuit
Source: Light Sci Appl. 2026 Jan 3;15:43. doi: 10.1038/s41377-025-02150-z (PMC12764773; doi:10.1038/s41377-025-02150-z)
Supplement: Supplementary file 1 — Supplementary Material [file 41377_2025_2150_MOESM1_ESM.pdf]

Supplementary Information for Programmable Bell State Generation in an Integrated Thin Film Lithium Niobate Circuit

Andreas Maeder[1,†,*], Robert J. Chapman[1,†], Alessandra Sabatti[1], Giovanni Finco[1], Jost Kellner[1], Rachel Grange[1]

[1]ETH Zurich, Department of Physics, Institute for Quantum Electronics, Optical Nanomaterial Group, Zurich, Switzerland

[†] These authors contributed equally to this work
[*]Corresponding author: maederan@phys.ethz.ch

CONTENTS

I Derivation of On-chip State Generation . . . 2

II Coincidence-to-accidental Ratio Characterization . . . 4

III Maximum Likelihood Estimation . . . 5

IV Full Experimental Results for State Generation . . . 7

V Setup and Characterization . . . 11
V.A Experimental Setup . . . 11
V.B Building Block Characterization . . . 12
V.C Experiment Stability . . . 16

VI Literature Comparison . . . 17

Supplementary References . . . 22

I DERIVATION OF ON-CHIP STATE GENERATION

Here, we discuss the generation and evolution of the state generated by the circuit shown in fig. 1a of the main manuscript. First, the input power P_0 is distributed by the Mach-Zehnder interferometer (MZI) controlled by the phase φ_1 into two modes A and B. The resulting optical power in each mode is

$$P_A = P_0 \sin^2\left(\frac{\varphi_1}{2}\right)$$
$$P_B = P_0 \cos^2\left(\frac{\varphi_1}{2}\right)$$

This assumes that the two directional couplers used as beamsplitters are behaving nominally with a reflectance $R = 0.5$. Besides any global phases omitted here, the two pump modes may acquire a relative phase θ_1 due to slight variations in the waveguide dimensions.

The entangled state is generated in the two periodically poled waveguides, which each create a two-mode squeezed vacuum state

$$|\psi_k\rangle = \sum_{n=0}^{\infty} \frac{[\tanh(r_k)]^n}{\cosh(r_k)} |n_s n_i\rangle_k$$

where $k = A, B$. The squeezing parameters r_k depend on the respective pump power and the spontaneous parametric down-conversion (SPDC) generation efficiency η_k, namely $r_k = \eta_k\sqrt{P_k}$. They are therefore implicitly controlled by the phase φ_1. In a low pump power regime, which we can always achieve by choosing P_0 sufficiently small, we can expand the infinite series up to first order and neglect any contributions $\mathcal{O}(r_k^2)$. This allows us to write the state generated by the combined two-mode system as

$$|\psi\rangle = |\psi\rangle_A \otimes |\psi_B\rangle = \Big(e^{i\theta_1}|0_s0_i\rangle_A + r_A e^{i\theta_1}|1_s1_i\rangle_A\Big) \otimes \Big(|0_s0_i\rangle_B + r_B|1_s1_i\rangle_B\Big)$$

The phase θ_1 is inherited by the SPDC photons since it is a coherent process. By expanding the tensor product we can further rewrite the state to

$$|\psi\rangle = e^{i\theta_1}|0_s0_i\rangle_A|0_s0_i\rangle_B + r_A e^{i\theta_1}|1_s1_i\rangle_A|0_s0_i\rangle_B + r_B|0_s0_i\rangle_A|1_s1_i\rangle_B + r_A r_B e^{i\theta_1}|1_s1_i\rangle_A|1_s1_i\rangle_B$$

Because of the low pump power regime the circuit operates in, we are safe to assume $r_A \cdot r_B \ll r_A, r_B$ and can therefore neglect the four-photon term. Furthermore, the vacuum state in the first term does not leave experimental signatures in coincidence measurements and can be dropped for the discussion here. This gives us the state generated after the periodically poled waveguides:

$$|\psi\rangle \approx r_A e^{i\theta_1}|1_s1_i\rangle_A|0_s0_i\rangle_B + r_B|0_s0_i\rangle_A|1_s1_i\rangle_B$$

The splitting of modes A, B into the four modes a, b, c, d and the subsequent crossing lead to the generation of the post-selected state

$$|\psi\rangle = r_A e^{i\theta_1} |1\rangle_a |0\rangle_b |1\rangle_c |0\rangle_d + r_B |0\rangle_a |1\rangle_b |0\rangle_c |1\rangle_d$$
$$\hat{=} r_A e^{i\theta_1} |00\rangle + r_B |11\rangle$$

where in the final step we used standard two-qubit state notations assuming that modes a, b and c, d each form a dual-rail encoded qubit. This state, up to the phase factor, forms the entangled Bell state Φ^+.

The phase θ_2 introduced by the thermo-optic phase shifter before the splitting controls this phase factor, and the MZI controlled by θ_2 adjust the state of the second qubit. We can include these phases in our final state:

$$|\psi\rangle = r_A e^{i(\theta_1+2\theta_2)} \left[\sin\left(\frac{\varphi_2}{2}\right)|00\rangle + \cos\left(\frac{\varphi_2}{2}\right)|01\rangle\right] + r_B \left[\cos\left(\frac{\varphi_2}{2}\right)|10\rangle - \sin\left(\frac{\varphi_2}{2}\right)|11\rangle\right].$$

Finally, to write the full dependence on thermo-optically controlled phases, we can include the implicit dependence on φ_1 through the squeezing parameters:

$$\begin{aligned}|\psi\rangle = \quad & \eta_a e^{i(\theta_1+2\theta_2)} \sin\left(\frac{\varphi_1}{2}\right)\sin\left(\frac{\varphi_2}{2}\right)|00\rangle \\ &+ \eta_a e^{i(\theta_1+2\theta_2)} \sin\left(\frac{\varphi_1}{2}\right)\cos\left(\frac{\varphi_2}{2}\right)|01\rangle \\ &+\eta_b \cos\left(\frac{\varphi_1}{2}\right)\cos\left(\frac{\varphi_2}{2}\right)|10\rangle \\ &-\eta_b \cos\left(\frac{\varphi_1}{2}\right)\sin\left(\frac{\varphi_2}{2}\right)|11\rangle .\end{aligned}$$

Note that we set $P_0 = 1$ since it is just a normalization constant. Also, we assumed perfect beamsplitters with $R = 0.5$ throughout this derivation. In the case of pumping with equal strengths, that is $\varphi_1 = \pi/2$, and assuming $\eta_a = \eta_b \equiv \eta$ we can rewrite the computational basis states as linear combinations of the Bell states, for example $|00\rangle = (|\Phi^+\rangle - |\Phi^-\rangle)/\sqrt{2}$, to arrive at this expression of the state in Bell basis:

$$\begin{aligned}|\psi\rangle = \quad & \frac{\eta}{\sqrt{2}}\left(e^{i(\theta_1+2\theta_2)} - 1\right)\sin\left(\frac{\varphi_2}{2}\right)|\Phi^+\rangle \\ &+\frac{\eta}{\sqrt{2}}\left(e^{i(\theta_1+2\theta_2)} + 1\right)\sin\left(\frac{\varphi_2}{2}\right)|\Phi^-\rangle \\ &+\frac{\eta}{\sqrt{2}}\left(e^{i(\theta_1+2\theta_2)} + 1\right)\cos\left(\frac{\varphi_2}{2}\right)|\Psi^+\rangle \\ &+\frac{\eta}{\sqrt{2}}\left(e^{i(\theta_1+2\theta_2)} - 1\right)\cos\left(\frac{\varphi_2}{2}\right)|\Psi^-\rangle\end{aligned}$$

By setting $\theta_1 = 0$, which can always be achieved by shifting the phase setting of θ_2 by $\theta_1/2$, and subsequently rewriting the exponentials as trigonometric functions, we arrive at the equation for the state in the main text.

II COINCIDENCE-TO-ACCIDENTAL RATIO CHARACTERIZATION

The coincidence-to-accidental ratio (CAR), defined as the ratio of the rate of true and accidental coincidences, $\mathrm{CAR} = R_{\mathrm{true}}/R_{\mathrm{acc}}$ is an important practical metric for experiments using photon pairs. It provides an upper bound on the measurable quantum interference visibility defined as

$$V = \frac{C_{\mathrm{dist}} - C_{\mathrm{indist}}}{C_{\mathrm{dist}}} \tag{S1}$$

where $C_{\mathrm{(in)dist}}$ are coincidences when photons are (in)distinguishable. In a continuous wave (CW) pumped experiment, when photons are distinguishable, we get contributions from true and accidental coincidences, $C_{\mathrm{dist}} = R_{\mathrm{true}} + R_{\mathrm{acc}}$, while for the perfectly indistinguishable case we only measure accidental coincidences, $C_{\mathrm{indist}} = R_{\mathrm{acc}}$. This gives an upper bound of the visibility

$$V = \frac{R_{\mathrm{true}}}{R_{\mathrm{true}} + R_{\mathrm{acc}}} = \frac{R_{\mathrm{true}}/R_{\mathrm{acc}}}{R_{\mathrm{true}}/R_{\mathrm{acc}} + 1} = \frac{\mathrm{CAR}}{\mathrm{CAR} + 1} \tag{S2}$$

For the quantum interference experiment reported in fig. 3c, the formula above can be inverted to get an estimated CAR between 57 and 300, considering the measured visibility of $99.0 \pm 0.7\,\%$ together with its uncertainty bounds.

To characterize the dependence of the CAR on the pair generation rate, we consider the signal, idler and coincidence counts [S1]:

$$N_s = R_{\mathrm{true}}\eta_s + d_s \tag{S3}$$

$$N_i = R_{\mathrm{true}}\eta_i + d_i \tag{S4}$$

$$N_{cc} = R_{\mathrm{true}}\eta_s\eta_i + N_{\mathrm{acc}} \tag{S5}$$

where $\eta_{s,i}$ are the signal and idler collection efficiency, $d_{s,i}$ is the dark count rate of the signal/idler detection channels, and $N_{\mathrm{acc}} = N_s N_i \Delta t_{\mathrm{bin}}$ is the number of accidental counts in a timebin Δt_{bin}. To simplify the algebraic manipulations, we consider the inverse of CAR and rewrite it:

$$\begin{aligned}
\mathrm{CAR}^{-1} &= \frac{N_{\mathrm{acc}}}{N_{\mathrm{cc}} - N_{\mathrm{acc}}} && \text{(S6)}\\
&= \frac{N_s N_i \Delta t_{\mathrm{bin}}}{R_{\mathrm{true}}\eta_s\eta_i} && \text{(S7)}\\
&= \frac{(R_{\mathrm{true}}\eta_s + d_s)(R_{\mathrm{true}}\eta_i + d_i)\Delta t_{\mathrm{bin}}}{R_{\mathrm{true}}\eta_s\eta_i} && \text{(S8)}\\
&= \left(R_{\mathrm{true}} + \frac{d_i}{\eta_i} + \frac{d_s}{\eta_s} + \frac{d_i d_s}{R_{\mathrm{true}}\eta_i\eta_s}\right)\Delta t_{\mathrm{bin}}. && \text{(S9)}
\end{aligned}$$

We rewrite the above equation by defining $a = d_i/\eta_i$ and $b = d_s/\eta_s$. Furthermore, we rescale $R_{\mathrm{true}} \to \alpha R_{\mathrm{true}}$ to take deviations of the measured PGR into account. We also assume equal

collection efficiency and dark count rates for signal and idler and can hence set $b = a$. This leaves us with the following fitting function

$$\mathrm{CAR}^{-1} = \left(\alpha R_{\mathrm{true}} + 2a + \frac{a^2}{\alpha R_{\mathrm{true}}}\right)\Delta t_{\mathrm{bin}}, \tag{S10}$$

which is used to produce the fit in fig. 2b of the main manuscript with a and α as the only fitting parameters and $\Delta t_{\mathrm{bin}} = 1\,\mathrm{ns}$. Note that the last term, which are accidental coincidences of one SPDC photon with a dark count photon, leads to the deviation from the linear behavior in the double logarithmic representation in fig. 2b. However, the reduction of the CAR for low PGR is well known [S1, S2].

Here, we also report the details for the two fits in fig. 2b of the manuscript. First, the fitting parameters for the filtered case are $\alpha = 1.80 \pm 0.02$, $a = 42.7 \pm 1.8\,\mathrm{kHz}$ and $\alpha = 2.77 \pm 0.04$, $a = 157.7 \pm 32.4\,\mathrm{kHz}$ in the unfiltered case. The fact that $\alpha > 1$ in both cases is an indication that the on-chip PGR could be underestimated. Furthermore, we report the usual coefficient of determination R^2 of 0.942 and 0.987, for the filtered and unfiltered fit, respectively.

III MAXIMUM LIKELIHOOD ESTIMATION

Reconstructing the full density matrix of a quantum state theoretically requires 16 measurements projecting the state onto different bases [S3]. Enabled by simultaneous coupling and measurement of both qubit rails with four independent single photon detectors, projections along the positive and negative direction of an axis is implemented without additional effort. Furthermore, the high pair generation rates of our on-chip sources lead to sufficiently short integration times, such that projecting onto all combinations of x, y and z axes for each qubit does not significantly increase the measurement duration. Therefore, we use the full set of 36 projection measurements to perform the density matrix reconstruction. This has been shown to improve performance when using maximum likelihood estimation [S4].

Experimentally, we perform full two-qubit tomography by implementing all pairs of local Pauli operators $\Pi_A, \Pi_B \in \{\sigma_x, \sigma_y, \sigma_z\}$ with the tomography part of the circuit. Each of the 9 combinations (Π_A, Π_B) is implemented using a specific phase setting $\Xi_\ell = (\varphi_{3,\ell}, \theta_{3,\ell}, \varphi_{4,\ell}, \theta_{4,\ell})$. For a given Ξ_ℓ we set the projection phases and record the coincidence counts $C_{jk,\ell}$ between output rails $j \in \{a, b\}$ of Q_A and $k \in \{c, d\}$ of Q_B. The values for $C_{jk,\ell}$ are obtained by summing the correlation histogram and subtracting accidental counts. Depending on the specific combination of qubit modes, a different combination of projections onto (P_A, P_B) is measured (see Fig. 3c of the

main manuscript). Since the measurements use four independent detectors, care has to be taken to consider varying detection efficiencies. Variations in coupling efficiencies or transmission losses in fiber filters are also lumped into this detection efficiency. Therefore, we use a slightly adjusted version of the standard MLE procedure detailed here.

The likelihood function used for MLE is

$$\mathcal{L}(\hat{\rho}) = \sum_{j=1}^{9} \sum_{\substack{j\in\{a,b\} \\ k\in\{c,d\}}} \left| C_{jk,\ell} - \tilde{C}(\hat{\rho}, \Pi_{jk,\ell}) \right|^2 \tag{S11}$$

where $C_{jk,\ell}$ are the measured coincidences and $\tilde{C}(\hat{\rho}, \Pi_{jk,\ell})$ is the theoretically expected number of coincidences for the state $\hat{\rho}$ under the two-qubit projector $\Pi_{jk,\ell}$. The expected coincidence counts are derived from the theoretical probability $p(\hat{\rho}, \Pi) = \mathrm{Tr}(\hat{\rho} \cdot \Pi)$ and normalized using

$$\tilde{C}(\hat{\rho}, \Pi_{jk,\ell}) = p(\hat{\rho}, \Pi_{jk,\ell}) \frac{\sum_{j=1}^{9} \sum_{\substack{j\in\{a,b\} \\ k\in\{c,d\}}} \mathcal{N}_{jk} C_{jk,\ell}}{\sum_{j=1}^{9} \sum_{\substack{j\in\{a,b\} \\ k\in\{c,d\}}} p(\hat{\rho}, \Pi_{jk,\ell})}. \tag{S12}$$

Here, we introduced additional normalization constants $\mathcal{N}_{jk}$ which take varying detection efficiencies for the different combinations of qubit modes into account. The density matrix $\hat{\rho}$ is parametrized as $\rho = T^\dagger T / Tr(T^\dagger T)$, where T is a lower-triangular matrix parametrized by 16 real

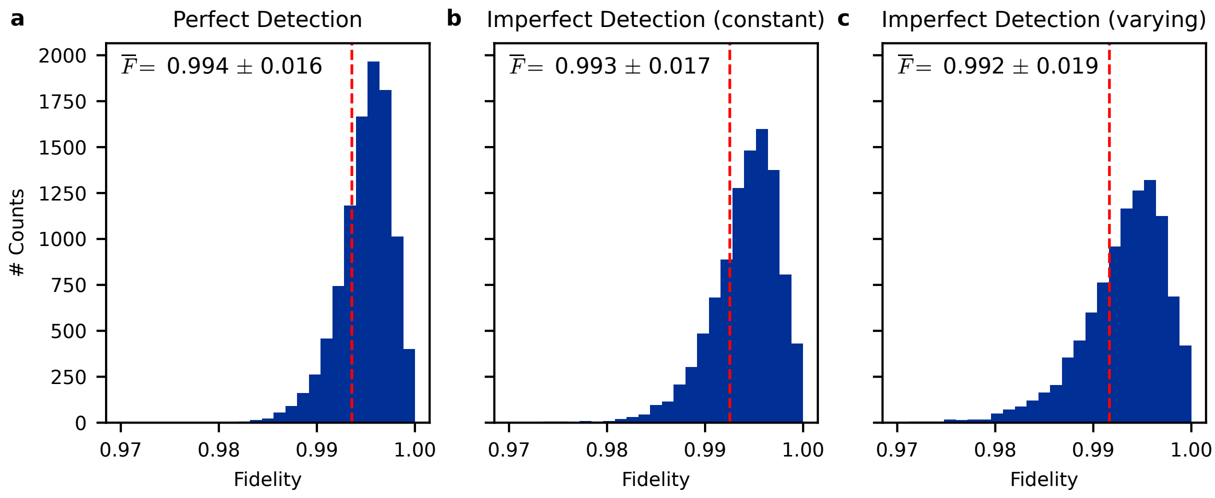

Figure S1. **Reconstruction algorithm performance under varying detection efficiency conditions.** Shown are histograms of fidelity values obtained from reconstructing 1000 random, synthetic quantum states per scenario using our algorithm. (**a**) Ideal case with perfect detection efficiency (100 %) for all qubit mode combinations. (**b**) Constant but non-uniform detection efficiency across the four combinations. (**c**) Randomly varying detection efficiency between individual measurements. For each case, the average fidelity $\overline{F}$ is indicated in the figure panel.

parameters and we use the four normalization constant as additional free parameters. We numerically minimize $\log[\mathcal{L}(\hat{\rho})]$ using the L-BFGS-B algorithm implemented in the SciPy package. The result of this minimization is the density matrix $\hat{\rho}_0$ which best describes the measurement results.

For verification of our implemented algorithm we use a synthetic sets of measurement data obtained by numeric simulation of the photonic circuit. We compare the ideal case, where the detection efficiency is equal at 100 % for each combination of qubit modes, with two non-ideal scenarios: one where the efficiency is less than 100 % and different for each of the four combinations but constant across the dataset, and another where it even varies between measurements in the same dataset. Each synthetic dataset consists of 1000 samples for which we reconstruct the density matrix using our algorithm and then report the fidelity to the initial state. Histograms of the fidelities are shown in fig. S1 alongside the mean fidelities $\overline{F}$. In all three cases we obtain a mean fidelity above 99 % with only a small deterioration along the datasets. We believe that this is due to the numerical precision of the optimizer used and not a limit of the algorithm.

IV FULL EXPERIMENTAL RESULTS FOR STATE GENERATION

Here, we report the raw count data used for the tomography of the four computational basis states as well as the four Bell states as bar plots in fig. S2. Together with the experimental data, we also indicate the expected coincidence counts $\tilde{C}$ (see eq. (S12)) for the best fit obtained by MLE. Both of these include the normalization factors $\mathcal{N}_{jk}$ introduced in supplementary material III. Since four projections are measured simultaneously, the generation rate of the target state can be obtained by summing the counts for four simultaneous measurements. The extracted generation rate R_{gen} indicated in fig. S2 for each state, is obtained by averaging the rate for each of the nine experimental settings used. With the exception of $|00\rangle$, we obtained rates above 1 kHz, which is the rate indicated in the main text. The theoretical counts shown in fig. S2 are obtained by multiplying the measurement probability for each projection by R_{gen}.

For completeness, we show the imaginary components of the reconstructed density matrices for the states presented in the main manuscript in fig. S3. For each state, we also provide the fidelity F, concurrence C, and the von Neumann entropies S_A and S_B of the reduced density matrices obtained by tracing out one qubit, as summarized in table S1. All metrics are computed using the QuTiP library in Python. Uncertainties are estimated as the standard deviation from Monte Carlo resampling of the experimentally measured counts, assuming Poissonian statistics, followed by maximum likelihood estimation for each sample.

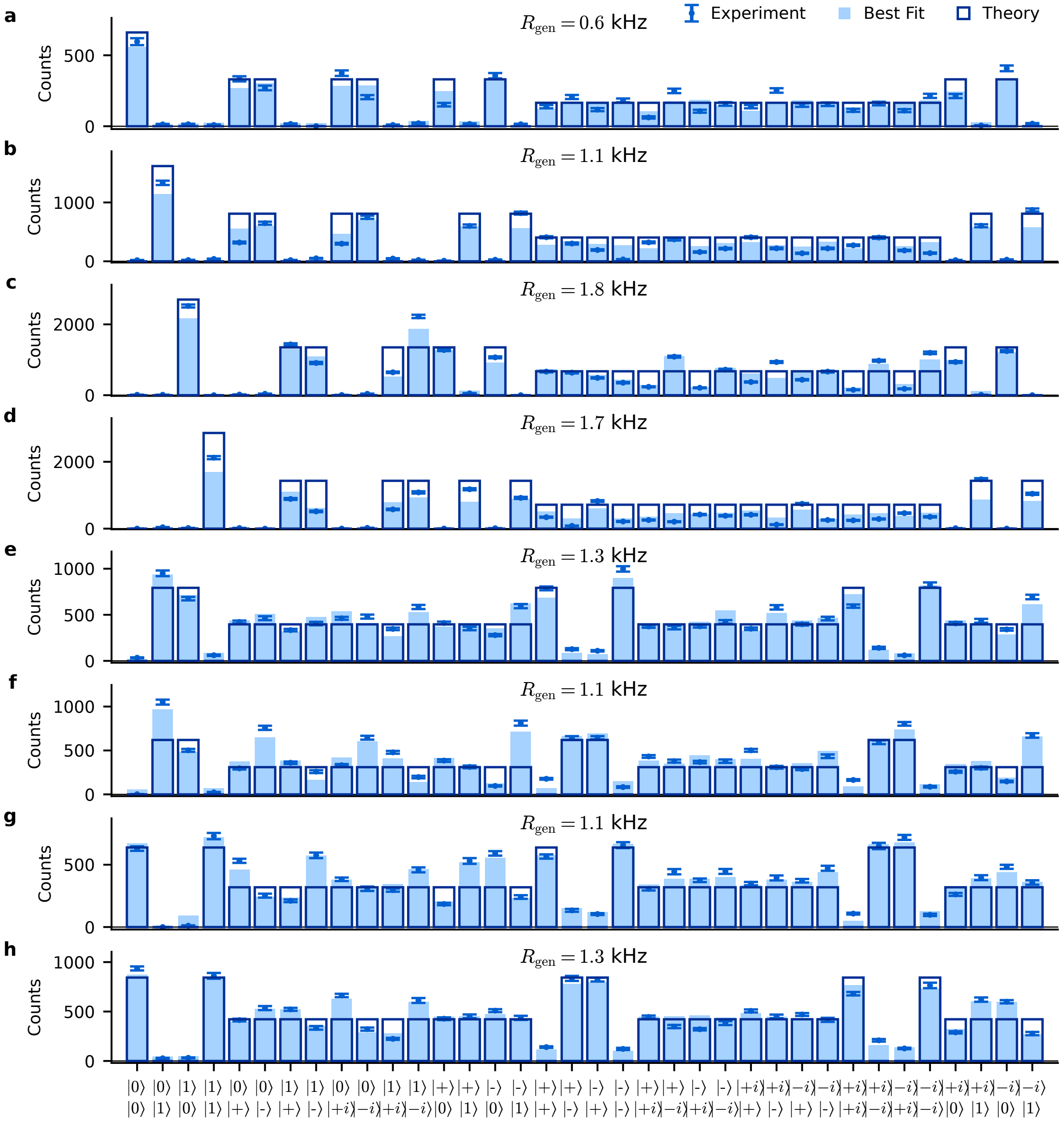

Figure S2. **Raw experimental data.** Raw experimental data used for state tomography for (**a**) $|00\rangle$, (**b**) $|01\rangle$, (**c**) $|10\rangle$, (**d**) $|11\rangle$, (**e**) $|\Psi^+\rangle$, (**f**) $|\Psi^-\rangle$, (**g**) $|\Phi^+\rangle$, and (**h**) $|\Phi^-\rangle$. Refer to the supplementary text for more details on the figure.

Table S1. Summary of the experimental results for the generation of target quantum states. The table lists the input phase settings φ_1, φ_2 and θ_2 used in the state preparation, along with the fidelity F, concurrence C, and von Neumann entropies S_A and S_B of the reduced density matrices obtained by tracing out one qubit. All values include one standard deviation uncertainty, estimated via Monte Carlo resampling.

Target State	φ_1	φ_2	θ_2	F [%]	C	S_A	S_B
$\|00\rangle$	π	0	π	95.1 ± 0.5	0.13 ± 0.03	0.25 ± 0.02	0.23 ± 0.2
$\|01\rangle$	π	0	0	98.4 ± 0.5	0.13 ± 0.02	0.09 ± 0.04	0.05 ± 0.02
$\|10\rangle$	0	0	π	95.0 ± 0.2	0.05 ± 0.01	0.006 ± 0.004	0.006 ± 0.003
$\|11\rangle$	0	0	0	98.7 ± 0.6	0.06 ± 0.02	0.017 ± 0.005	0.014 ± 0.004
$\|\Psi^+\rangle$	$\pi/3$	0	0	93.1 ± 0.6	0.8 ± 0.03	0.682 ± 0.005	0.67 ± 0.01
$\|\Psi^-\rangle$	$\pi/3$	0	$\pi/2$	90.8 ± 0.7	0.8 ± 0.02	0.64 ± 0.01	0.64 ± 0.01
$\|\Phi^+\rangle$	$\pi/3$	π	$\pi/2$	90.0 ± 0.6	0.73 ± 0.02	0.688 ± 0.004	0.689 ± 0.003
$\|\Phi^-\rangle$	$\pi/3$	π	0	91.1 ± 0.6	0.74 ± 0.02	0.692 ± 0.001	0.693 ± 0.001

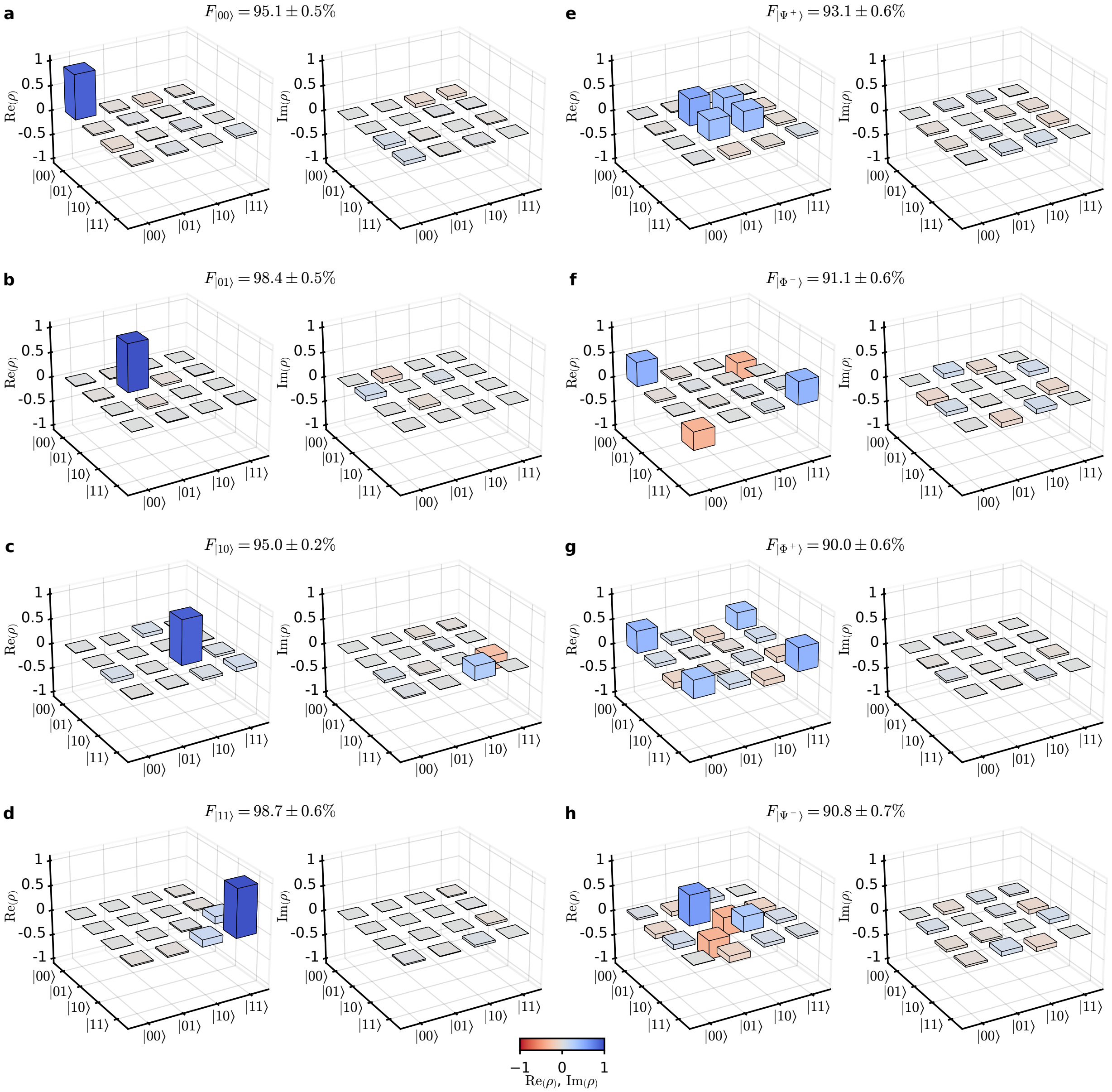

Figure S3. **Full Reconstructed Density Matrices.** Real and imaginary parts of the density matrices obtained via maximum likelihood estimation (MLE) for the states (**a**) $|00\rangle$, (**b**) $|01\rangle$, (**c**) $|10\rangle$, (**d**) $|11\rangle$, (**e**) $|\Psi^+\rangle$, (**f**) $|\Psi^-\rangle$, (**g**) $|\Phi^+\rangle$, and (**h**) $|\Phi^-\rangle$. These plots provide a complete representation of the reconstructed two-qubit states, complementing the real-part-only visualizations shown in the main text. Fidelities $F_{|\psi\rangle}$ refer to the overlap between the reconstructed density matrix and the respective target state $|\psi\rangle$.

V SETUP AND CHARACTERIZATION

V.A Experimental Setup

The experimental setup used for the photon pair source measurements is shown in fig. S4a. A continuous wave (CW) laser at $\lambda = 775\,\text{nm}$ is coupled to the device-under-test using single mode fiber and grating couplers. A manual polarization controller is used to adjust the polarization to maximum transmission which corresponds to exciting the TE_0 mode. To monitor the input power, a 99:1 fiber splitter diverts 1 % of the light to the chip while sending the remaining 99 % to a power meter. After out-coupling of the SPDC signal into a single mode fiber, it is filtered using a longpass filter, split into two paths using a 50:50 fiber beamsplitter and detected using two superconducting nanowire single photon detectors (SNSPDs). Prior to each SNSPD, an additional manual polarization controller is used to adjust the polarization of the signal for maximum detection efficiency, which is 80 % as specified by the commercial supplier. For the measurements where the photon pair signal was spectrally filtered, an additional 2 nm bandpass filter is inserted prior to the longpass filter. To measure the single photon spectra (fig. 2c of the manuscript), a dispersion compensation module is inserted into one path. This introduces a wavelength dependent delay of about 0.5 ns/nm.

Figure S4b shows a schematic of the experimental setup used for the two-qubit state generation measurements. While the input side is the same as described above, we use fiber arrays for out-coupling. As discussed in the Methods section, due to limited availability of bandpass filters, only the first qubit is spectrally narrowed. However, because of the energy conserving nature of the SPDC process, and the post-selection to events with one photon per qubit, this effectively also filters the second qubit. A multi-channel voltage source capable of supplying up to 600 mW per channel is connected to the PCB which is wirebonded to the photonic chip. Custom Python scripts are used to control the entire experiment.

On top of the fiber-to-chip coupling loss (see supplementary material V.B), the longpass filters introduce approximately 0.4 dB of additional loss, while the bandpass filters contribute about 1.5 dB of loss. We assume around 1 dB of additional transmission loss due to fiber propagation and interface loss and a detection efficiency of 80 % for all SNSPD channels as per the manufacturer specifications. Since this should be understood as an upper bound, the inferred on-chip rates are worst-case estimates with respect to the single photon detection efficiencies.

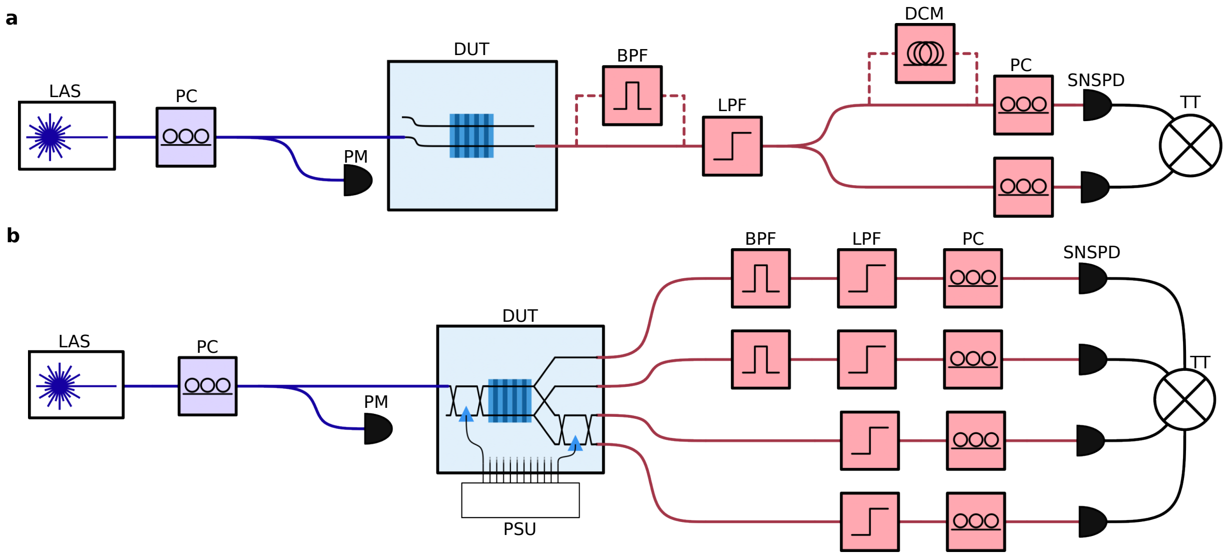

Figure S4. **Measurement setups.** Experimental setups used for (**a**) SPDC source characterization and (**b**) two-qubit state generation and tomography measurements. Abbreviations: LAS: Laser; PC: polarization controller; PM: power meter; DUT: device-under-test; PSU: power supply unit; BPF: bandpass filter; LPF: longpass filter; SNSPD: superconducting nanowire single photon detector; TT: time tagger; DCM: dispersion compensation module.

V.B Building Block Characterization

To confirm the characteristics of important building blocks, we fabricated copies of relevant devices on the same chip and measured their properties. We used grating couplers connected by a straight waveguide to determine the coupling losses both at the SPDC wavelength 1550 nm and the pump wavelength of 775 nm. Spectral response measurements corrected for measurement system losses are shown in fig. S5a and fig. S5b, respectively. The measured coupling loss at 1550 nm is 6.2 dB per grating and at 775 nm it is 10.2 dB per grating. Both of these values are above current state-of-the-art grating couplers used on the LNOI platform [S5]. This is in part due to the fact that we are using a SiO_2 cladding layer, which reduces the index contrast and therefore the diffractive strength of the gratings. Since the SPDC sources require sub-mW pump powers, the losses on the pump side pose no experimental limitation as we use a laser with 20 mW power. On the single photon side, the losses are limiting achievable pair detection rates. However, since we still obtain coincidence counts in the kHz range, this is sufficient to perform experiments with reasonably low integration times.

In the response of the 775 nm grating couplers, we observe distinct oscillations spaced by around

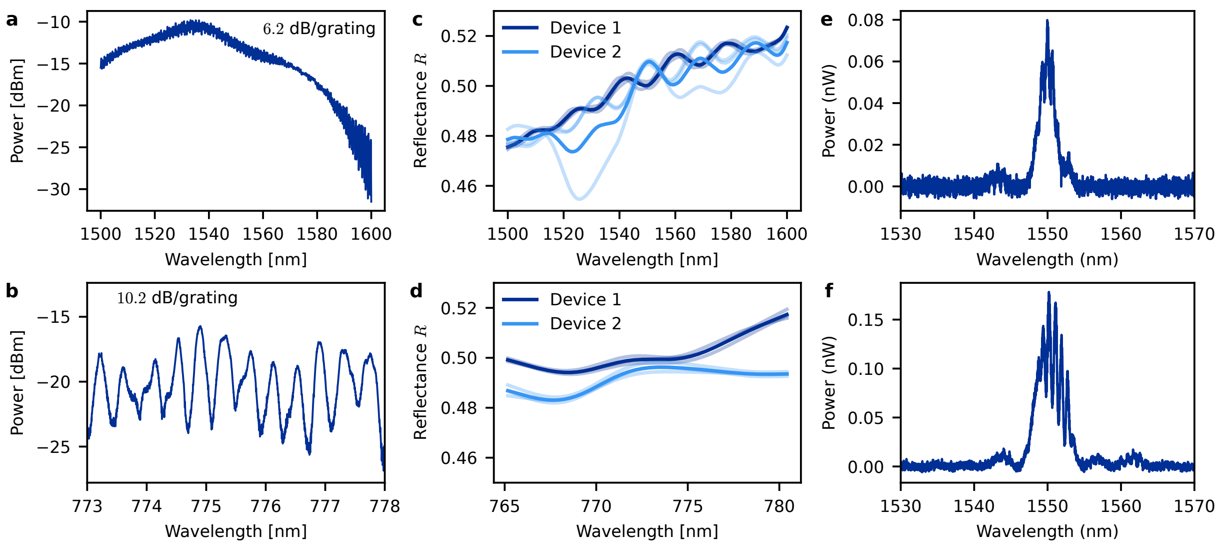

Figure S5. **Experimental characterization of generator circuit building blocks.** Spectral characterization of grating couplers designed for wavelengths around (**a**) 1550 nm and (**b**) 775 nm with coupling loss per grating indicated in the respective panel. Reflectance of on-chip directional couplers for two separate devices designed for (**c**) 1550 nm and (**d**) 775 nm. Second harmonic measurements for (**e**) source A and (**f**) source B of the Bell state generator circuit.

400 pm. These arise from reflections between the on-chip gratings which form a Fabry-Pérot cavity. Although less pronounced, the 1550 nm grating show similar oscillations for the same reason. Both of these spectral responses are the source of the observed oscillations in the second harmonic response in fig. 2a,b of the manuscript. Additionally, they account for the observed second harmonic efficiency difference between the two sources, as the oscillations are relatively pronounced and the oscillation peak positions depend on fiber alignment.

For the performance of the on-chip Mach-Zehnder interferometers (MZIs), the reflectance R of the directional couplers has to be as close to 0.5 as possible. We measure this on two copies of the designed directional couplers using a method relying on transmission measurements of a single directional coupler to get its reflectance [S6]. The results for directional couplers designed for 1550 nm (fig. S5c) and 775 nm (fig. S5d) show $R \sim 0.5$ around the design wavelength in both cases. The oscillations observed are believed to be due to parasitic reflections of the gratings (see fig. S5a,b).

Finally, fig. S5e and f show second harmonic spectra obtained by pumping the Bell state generation circuit backwards. These spectra confirm phase matching around 1550 nm, which matches the results obtained from the calibration sources reported in the main text. None of the phase shifters

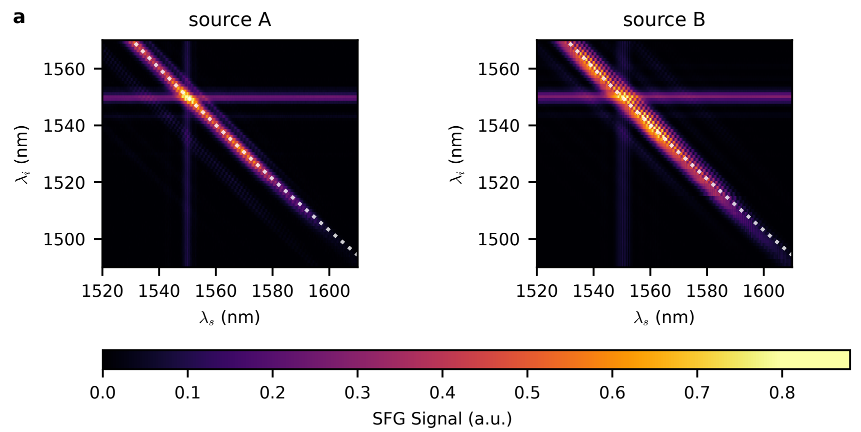

Figure S6. **Sum Frequency Generation (SFG) measurements.** SFG signal of SPDC source A and B as a function of signal and idler wavelengths $\lambda_{s,i}$. The white dotted line indicates the energy conservation of the SPDC process.

to optimize transmission and absolute power were used during these measurements. The spectral shape is therefore highly dependent on the on-chip components light is passing through. Note that this is not the same for the two calibration sources, hence the observed differences are expected. Nevertheless, the almost identical phase matching wavelength to the test waveguides (see fig. 2a) shows, that, although the phase matching is highly sensitive to film thickness variations [S7], our film is uniform enough over the distance between the generator and test waveguide poling regions, which is around 600 µm. This justifies our choice of using a single poling period instead of adapting it along the poled waveguides as in [S7]. Using this approach, we observed deviations of the phase matching wavelength no bigger than 10 nm from the target wavelength for different poling sections across the $15 \times 15\,\mathrm{mm}^2$ LNOI chip.

To characterize the type-0 SPDC process further, we also performed sum frequency generation (SFG) measurements, which probe the same phase matching function that governs the SPDC process. For this, we coupled two tunable CW lasers to a single fiber which we used to excite both sources by pumping the circuit backwards and collecting the SFG signal around 775 nm. The resulting maps when sweeping the signal and idler wavelengths are shown in fig. S6 for both sources. As is expected for a type-0 process, the phase matching function is very broadband and in our case limited by the grating response (see fig. S5a) of the 1550 nm gratings. We attribute the observed difference in the width of the response, which is also shown in the SHG measurements in fig. S5e,f, to imperfections in the poling process. On top of the measured phase matching function we plot the pump envelope function which is determined by the energy conservation of the SPDC

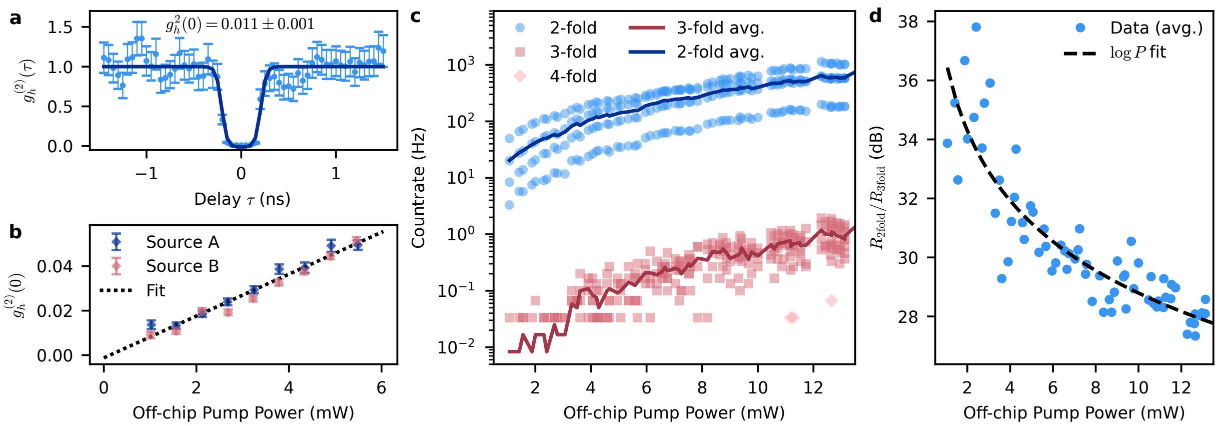

Figure S7. **Multi-pair generation properties of SPDC sources.** (**a**) Heralded second order correlation function $g_h^{(2)}$ of a filtered SPDC source pumped at 1.6 mW (off-chip). (**b**) $g_h^{(2)}(0)$ values for different off-chip applied pump powers. (**c**) Measured count rates of 2-fold, 3-fold and 4-fold coincidences when sweeping the pump power. (**d**) Suppression of 3-fold coincidences as compared to 2-folds. A logarithmic dependence of the pump power is fitted to the data (see main text for more details).

process, since we are pumping with a monochromatic source. Although this shows that the photon pairs are spectrally correlated, this does not pose a problem, since we do not use heralding in our experiment, but rely on the full two-photon state.

Since the state generated in the periodically poled waveguides is a squeezed vacuum (see supplementary material I), there is a non-zero probability of having higher order SPDC events. However, these are exponentially suppressed. We provide evidence for this in the form of heralded second order correlation measurements performed on the test sources using the setup shown in fig. S4a with an additional 50:50 beamsplitter on one of the photons. We refer to existing literature for an in depth discussion of the measurement and shape of $g_h^{(2)}(\tau)$ [S8, S9]. Figure S7a shows a full $g_h^{(2)}(\tau)$ measurement for an off-chip pump power similar to the one used in the tomography experiment, while fig. S7b shows the value at zero delay for different pump powers. In the latter, the expected linear dependence on the pump power is observed.

For a more direct investigation of potential contamination due to higher order SPDC events, we measured 2-, 3- and 4-fold coincidence events in all possible combinations of the four outputs using the setup shown in fig. S4b. The results shown in fig. S7c indicate an average suppression of three orders of around magnitude of 2- fold coincidences as compared to 3- and 4-fold events. Note that this was an initial characterization measurement for which the coupling was not yet fully optimized, hence the lower coincidence count rate as compared to the final experiments (see supplementary

material IV). We extract the pump power dependence of the suppression $R_{2\text{fold}}/R_{3\text{fold}}$, where the rates are taken as the average rate over the respective channel combinations. We report this suppression in dB in fig. S7d, where we observe a logarithmic dependence on the pump power. This is entirely due to the conversion into dB-units and corresponds to a linear dependence on the pump power P. This is expected as $R_{2fold} \propto P$ and $R_{3\text{fold}} \propto P^2$. Given this level of suppression, at a 1 kHz coincidence count rate, we would expect a rate of about 1 Hz to be attributed to higher order SPDC events. This low rate is one of the advantages of the CW pumping scheme, where the absence of high peak powers significantly reduces the likelihood of multi-photon pair generation compared to pumping with pulsed lasers.

V.C Experiment Stability

To evaluate the stability of the two-qubit state generation experiment over time, we prepared the same two-qubit state 100 times and performed full state tomography on each instance. A waiting time of 30 s between successive tomographies was included, during which all the heaters were turned off. Importantly, we did use the same calibration dataset for all 100 state tomographies. For each measurement, the density matrix of the quantum state was reconstructed using the MLE algorithm described in supplementary material III. To quantify temporal stability, the density matrix obtained from the initial measurement was taken as the reference state, and the fidelity of all subsequent density matrices with respect to this reference was computed. The results, shown in fig. S8, indicate that the fidelity remains higher than 99 %, except for a few outliers. This demonstrates that our device supports stable operation over extended periods of time without the need for recalibration.

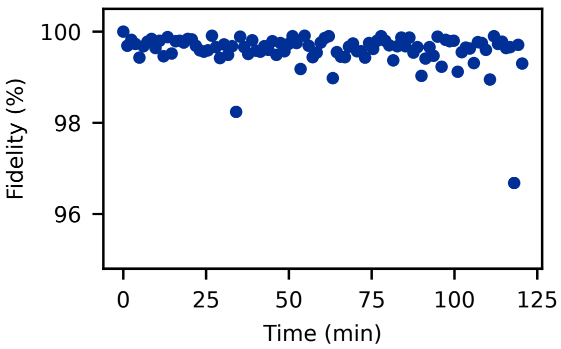

Figure S8. **Stability of state tomography experiment over time.** Fidelity of 100 successive state preparation and tomography experiments with respect to the initial state.

VI LITERATURE COMPARISON

When comparing our results to existing literature, two complementary perspectives should be considered: First, our on-chip source characteristics give insight into intrinsic device efficiency and fabrication quality. On the other hand, in the actual state preparation experiments, experimental metrics such as measured generation rates, required off-chip laser power and achieved fidelities including all the experimental imperfections are of much more interest. To cover both these aspects, tables S2 and S3 provide a comparative overview of the respective recent literature.

Table S2. **Comparison of integrated nonlinear photon pair sources.** Summary of intrinsic source characteristics including device type, nonlinear interaction length, brightness and measured CAR across different material platforms. Note the different units for the brightness values for SPDC- and SFWM-based sources as discussed in this supplementary section. Abbreviations: WG: waveguide; MR: microresonator; CAR: coincidence-to-accidental ratio; SFWM: spontaneous four-wave mixing; SPDC: spontaneous parametric down-conversion; PE-LN: proton-exchanged lithium niobate; LNOI: lithium niobate-on-insulator; N/A: not available.

**Source**	**Pump**	**Process**	**Length [mm]**	**Brightness [MHz/mW$^{(2)}$]**	**Spectral Brightness [MHz/mW$^{(2)}$/nm]**	**CAR**	**Ref.**
Si MR	CW	SFWM	-	149	1900	12105	[S10]
Si MR	CW	SFWM	-	5.3	133	602	[S11]
Si WG	CW	SFWM	10	0.076	19	673	[S12]
Si WG	CW	SFWM	25	100	126	140	[S13]
SiN MR	CW	SFWM	-	0.52	145	400	[S2]
SiN MR	CW	SFWM	-	30.3	146000	1483	[S14]
SiN WG	Pulsed	SFWM	65	0.018	0.022	16	[S15]
SiN WG	CW	SPDC[a]	81	0.015	0.0005	1635	[S16]
AlGaAs WG	CW	SPDC	N/A	11.5	1.07	141	[S17]
AlGaAs WG	Pulsed	SPDC	2	0.00075	0.00006	20	[S18]
PE-LN WG	CW	SPDC	10	200	14	N/A	[S19]
LNOI WG	CW	SPDC	5	45	56	67224	[S20]
LNOI WG	CW	SPDC	0.3	0.036	0.25	6900	[S21]
LNOI WG	CW	SPDC	1.5	56	26	14110	This Work

[a] Uses effective $\chi^{(2)}$ induced by all-optical poling.

Focusing on device level metrics, we compare our work to integrated nonlinear photon pair sources. Depending on the crystal symmetry, either spontaneous parametric down-conversion (SPDC, for non-centrosymmetric materials) or spontaneous four-wave mixing (SFWM, for centrosymmetric materials) is used. Note that the latter scales quadratically with the pump power P_p, hence the brightness values are normalized to P_p^2 and its unit is $\mathrm{MHz\,mW^{-2}}$, as compared to the linear pump power dependence of SPDC which leads to the unit $\mathrm{MHz\,mW^{-1}}$. Therefore, the suitability of brightness as a metric to compare SPDC- and SFWM-based sources is limited. However, based on the brightness, we can obtain the expected pair generation rate at $P_p = 1\,\mathrm{mW}$. If this rate is the same for an SPDC- and SFWM-based source, the SPDC will outperform the SFWM for $P_p < 1\,\mathrm{mW}$ in terms of pump efficiency due to the differences in pump power dependence. For $P_p > 1\,\mathrm{mW}$ the opposite is the case. Since on-chip pump powers are typically below 1 mW for efficient sources on various platforms, SPDC is expected to be more efficient relative to SFWM.

As shown in table S2, photon pair sources in silicon or silicon nitride can achieve higher brightness than what we report in this work. To achieve this, either micro-resonator or long waveguide spirals are employed. Micro-resonators add considerable design and fabrication complexity because coupling must be carefully engineered at both the pump and SFWM wavelengths. Spiral waveguides, in contrast, are straightforward to design but substantially increase the source footprint. This work, along with previous demonstrations of LNOI SPDC sources [S20], shows that comparable brightness can be achieved with an order of magnitude shorter waveguides.

Note that we only include our filtered brightness in table S2, as most studies also use off-chip filtering. Our highest unfiltered source brightness of $1.7\,\mathrm{GHz\,mW^{-1}}$ is almost one order of magnitude higher than the $230\,\mathrm{MHz\,mW^{-1}}$ reported in [S22].

When comparing this work to other demonstrations of on-chip generation and tomography of two-qubit entangled states in table S3, we report the lowest pump power for a continous-wave (CW) experiment. Furthermore, the generation rate of 1.3 kHz is an order of magnitude improvement compared to the work in Si or SiN, with the exception of [S23]. Given the high coupling losses of our grating couplers (see supplementary material V.B), there is a straightforward path for boosting our measured generation rate.

We include timebin encoded experiments in table S3 for completeness, although they are not directly comparable to our path-encoded approach. In timebin circuits, the relative optical phases between photons are inherently fixed by the interferometer geometry and are not adjustable. Path-encoded circuits like ours offer independent phase control of all qubits through a reconfigurable

on-chip interferometric network. Moreover, in timebin encoding based on Franson interferometers, photon indistinguishability originates from the pump interferometer. In contrast, in our CW-pumped approach, indistinguishability arises from the coherent superposition of emissions from two spatial modes. Furthermore, the rates observed in timebin experiments are limited by the low pump power required to suppress multi photon events, which is not a limiting factor in CW pumped experiments (see also supplementary material V.B). These differences show that path and timebin encoding are complementing techniques.

Table S3. **Comparison of on-chip Bell state generation experiments.** Only demonstrations with both on-chip source and analysis circuitry are included. Besides the material platform, source type and qubit encoding used, the measured state generation rate, the TPI visibility and the Bell state fidelity are reported. Abbreviations: SOI: silicon-on-insulator; LNOI: lithium niobate-on-insulator; SiN: silicon nitride; SFWM: spontaneous four-wave mixing; SPDC: spontaneous parametric down-conversion; TPI: two-photon interference.

**Platform**	**Source**	**Encoding**	**Off-chip pump power**	**Rate [kHz]**	**TPI visibility [%]**	**Fidelity [%]**	**Ref.**
SOI	SFWM	Path	$150\,\mu W^{a}$	0.03	95.8	91.1	[S24]
SOI	SFWM	Path	$30\,mW^{b,c}$	0.12^{c}	93.2	90.9	[S25]
SOI	SFWM	Path	$0.5\,mW^{a}$	15	98.7	98.9	[S23]
LNOI	SPDC	Timebin	$10\,\mu W^{a}$	0.005	-	91.9	[S26]
SiN	SFWM	Timebin	$5.7\,mW^{a,c}$	0.007	-	91.0	[S27]
LNOI	SPDC	Path	$1.7\,mW^{b}$	1.3	99.0	93.1	This Work

^{a} Pulsed ^{b} CW ^{c} Estimated from reported data

SUPPLEMENTARY REFERENCES

[S1] Zhang, Z. C. *et al.* High-performance quantum entanglement generation via cascaded second-order nonlinear processes. *npj Quantum Information* **7**, 123 (2021).

[S2] Samara, F. *et al.* High-rate photon pairs and sequential Time-Bin entanglement with Si_3N_4 microring resonators. *Optics Express* **27**, 19309–19318 (2019).

[S3] Altepeter, J. B., James, D. F. V. & Kwiat, P. G. 4 qubit quantum state tomography. In *Quantum State Estimation* (eds Paris, M. & Řeháček, J.) (Berlin, Heidelberg: Springer, 2004), 113–145.

[S4] de Burgh, M. D. *et al.* Choice of measurement sets in qubit tomography. *Physical Review A: Atomic, Molecular, and Optical Physics* **78**, 052122 (2008).

[S5] Lomonte, E. *et al.* Scalable and efficient grating couplers on low-index photonic platforms enabled by cryogenic deep silicon etching. *Scientific Reports* **14**, 4256 (2024).

[S6] Piasetzky, J. *et al.* Robust characterization of integrated photonics directional couplers (2024). 2412.11670v1.

[S7] Chen, P.-K. *et al.* Adapted poling to break the nonlinear efficiency limit in nanophotonic lithium niobate waveguides. *Nature Nanotechnology* **19**, 44–50 (2024).

[S8] Bettelli, S. Comment on "Coherence measures for heralded single-photon sources". *Physical Review A* **81**, 037801 (2010).

[S9] Guo, X. *et al.* Parametric down-conversion photon-pair source on a nanophotonic chip. *Light: Science & Applications* **6**, e16249–e16249 (2017).

[S10] Ma, C. *et al.* Silicon photonic entangled photon-pair and heralded single photon generation with CAR $>$ 12,000 and $g^{(2)}(0) < 0.006$. *Optics Express* **25**, 32995–33006 (2017).

[S11] Engin, E. *et al.* Photon pair generation in a silicon micro-ring resonator with reverse bias enhancement. *Optics Express* **21**, 27826–27834 (2013).

[S12] Guo, K. *et al.* High coincidence-to-accidental ratio continuous-wave photon-pair generation in a grating-coupled silicon strip waveguide. *Applied Physics Express* **10**, 062801 (2017).

[S13] Shin, W. *et al.* Photon-pair generation in a lossy waveguide. *Nanophotonics* **12**, 531–538 (2023).

[S14] Chen, R. *et al.* Ultralow-Loss Integrated Photonics Enables Bright, Narrowband, Photon-Pair Sources. *Physical Review Letters* **133**, 083803 (2024).

[S15] Zhang, X. *et al.* Correlated photon pair generation in low-loss double-stripe silicon nitride waveguides. *Journal of Optics* **18**, 074016 (2016).

[S16] Dalidet, R. *et al.* Near perfect two-photon interference out of a down-converter on a silicon photonic chip. *Optics Express* **30**, 11298–11305 (2022).

[S17] Autebert, C. *et al.* Integrated AlGaAs source of highly indistinguishable and energy-time entangled photons. *Optica* **3**, 143–146 (2016).

[S18] Chen, H. *et al.* Invited Article: Time-bin entangled photon pairs from Bragg-reflection waveguides. *APL Photonics* **3**, 080804 (2018).

[S19] Jin, H. *et al.* On-Chip Generation and Manipulation of Entangled Photons Based on Reconfigurable Lithium-Niobate Waveguide Circuits. *Physical Review Letters* **113**, 103601 (2014).

[S20] Zhao, J. *et al.* High quality entangled photon pair generation in periodically poled thin-film lithium niobate waveguides. *Physical Review Letters* **124**, 163603 (2020).

[S21] Elkus, B. S. *et al.* Generation of broadband correlated photon-pairs in short thin-film lithium-niobate waveguides. *Optics Express* **27**, 38521–38531 (2019).

[S22] Chapman, R. J. *et al.* On-chip quantum interference between independent lithium niobate-on-insulator photon-pair sources. *Physical Review Letters* **134**, 223602 (2025).

[S23] Paesani, S. *et al.* Near-ideal spontaneous photon sources in silicon quantum photonics. *Nature Communications* **11**, 2505 (2020).

[S24] Silverstone, J. W. *et al.* Qubit entanglement between ring-resonator photon-pair sources on a silicon chip. *Nature Communications* **6**, 7948 (2015).

[S25] Santagati, R. *et al.* Silicon photonic processor of two-qubit entangling quantum logic. *Journal of Optics* **19**, 114006 (2017).

[S26] Finco, G. *et al.* Time-bin entangled Bell state generation and tomography on thin-film lithium niobate. *npj Quantum Information* **10**, 135 (2024).

[S27] Zhang, X. *et al.* Integrated silicon nitride time-bin entanglement circuits. *Optics Letters* **43**, 3469–3472 (2018).
